# Accuracy of anthropometric indicators of obesity to identify high blood pressure in adolescents—systematic review

**DOI:** 10.7717/peerj.13590

**Published:** 2022-08-09

**Authors:** Leandro Lima Borges, Tiago Rodrigues de Lima, Diego Augusto Santos Silva

**Affiliations:** Universidade Federal de Santa Catarina, Florianópolis, Santa Catarina, Brasil

**Keywords:** Hypertension, Young adult, Precision, Body weight, Anthropometric indicators, High blood pressure

## Abstract

**Background:**

Anthropometric indicators of obesity have been associated with blood pressure in adolescents. However, the accuracy of anthropometric indicators of obesity for screening for high blood pressure (HBP) in adolescents is not known. Thus, the aim of the present study was to summarize the set of evidence regarding the predictive ability of anthropometric indicators of obesity to identify HBP in adolescents.

**Methods:**

Searches were performed in five databases: MEDLINE, Web of Knowledge, Scopus, Scientific Electronic online (SciELO) and SportDiscus. The inclusion criteria for studies were: adolescents aged 10–19 years or mean age included in this range, observational and intervention studies, studies that proposed cutoff points for anthropometric indicators of obesity, and studies in English, Portuguese and Spanish. The methodological quality of studies was assessed using the QUADAS-2 instrument.

**Results:**

Ten studies met the inclusion criteria and had their information summarized. Based on the information described in these studies, the anthropometric indicators body mass index (BMI), waist circumference (WC), waist-to-height-ratio (WHtR), triceps skinfold thickness, body adiposity index, C index, body mass, waist-to-arm span ratio, arm fat area, average arm perimeter, fat percentage and arm span were likely to be used in high blood pressure (HBP) screening among adolescents. However, only one study showed acceptable values (moderate to high precision) in relation to the accuracy measurements of described cutoffs.

**Conclusion:**

Caution is suggested in the use of anthropometric indicators of obesity for HBP screening in adolescents, in which a greater number of studies with accurate diagnostic tools are necessary.

## Introduction

Anthropometric indicators such as body mass (BM), height, waist circumference (WC) and hip circumference (HC) have been described as useful tools to detect factors associated with cardiovascular risk (Cassiano et al., 2019), such as insulin resistance, metabolic syndrome, and dyslipidemia (De Faria et al., 2009; Mastroeni et al., 2019; Beck, Da Silva Lopes & Pitanga, 2011). Additionally, the predictive power of anthropometric indicators for screening for high blood pressure (HBP) in children and adolescents (De Quadros et al., 2019; Rimárová et al., 2018) has been described, and it has been reported that anthropometric indicators of obesity such as body mass index (BMI), waist-to-height ratio (WHtR) and WC have acceptable discriminatory power to identify HBP in adolescents (Araújo, Ramos & Barros, 2019; Liew et al., 2019; De Araújo Pinto et al., 2017).

Although the measurement of blood pressure levels is an important component of health assessment routines in the pediatric population, difficulties related to the measurement (*e.g.*, need to use specific instrument or choose the appropriate cuff for the child/adolescent’s arm) or classification of measured information (*e.g.*, insertion of pressure values in panels of growth curves) are configured as barriers for the measurement of blood pressure levels in environments with structure different from that observed in clinical centers, such as schools or sports clubs (Barroso et al., 2021). Thus, the use of anthropometric indicators can be a useful tool in the screening for HBP in children and adolescents.

In this sense, the aim of this review was to investigate the accuracy of anthropometric indicators of obesity for screening for HBP in adolescents.

## Material & Methods

The method used in this systematic review was consistent with the Preferred Reporting Items for Systematic Reviews and Meta-Analyses (PRISMA) (Liberati et al., 2009) statement (Supplemental 1). The review was registered in the “International Prospective Register of Systematic Reviews” (PROSPERO) under number CRD42020151554, and is available in full at: http://www.crd.york.ac.uk/prospero/.

### Eligibility criteria

The inclusion criteria for studies were: (1) adolescents aged 10–19 years or evaluated with mean age of 10–19 years; (2) observational and intervention studies; (3) studies that proposed cutoff points for anthropometric indicators of obesity to predict HBP, high systolic (SBP) and/or diastolic (DBP) blood pressure (measured or self-reported); (4) studies in English, Portuguese and Spanish. As exclusion criteria: duplicate articles excluded by the system and manually (possible titles not automatically excluded by the software), review articles, monographs, dissertations or theses, abstracts, book chapters, points of view/opinions of experts, articles in which the sample size/population was composed only of athletes or individuals with health problems, studies in which there was no proposal of cutoff points for anthropometric indicators of obesity for the prediction of HBP considering the age range included in this review.

### Data sources and searches

The search for articles was carried out during the months of December 2019 and January 2020. The systematic search for information regarding the use of anthropometric indicators of obesity in order to propose cutoff points for HBP in adolescents was carried out in MEDLINE database by PubMed website, Web of Knowledge, Scopus, Scientific Electronic online (SciELO) and the SPORTDiscus database by the EBSCOhost website.

### Selection of studies

The selection process was carried out by two reviewers independently (LLB & TRL). Articles that did not meet the inclusion criteria after reading titles and abstracts were excluded. The remaining studies were read in full and were selected based on inclusion criteria. In case of doubts among researchers about the inclusion of articles, a senior researcher was consulted (DASS). After the selection of articles included in the review, reference lists were read with a view of identifying possible studies not identified in the systematic search.

The survey results in each database were exported to the EndNote® version X4 reference manager (Thomson ISI ResearchSoft, Clarivate Analytics, Philadelphia, PA, USA, 2010).

### Search strategies, descriptors and keywords

The selection of descriptors occurred by consulting the Health Sciences Descriptors (DeCS) (Pellizzon, 2004) and Medical Subject Headings (MeSH) platforms  (Dhammi & Kumar, 2014). In addition, terms, and keywords used in literature reviews and original articles were used to determine these descriptors. Thus, searches for available information were conducted considering studies in English, Spanish and Portuguese (Supplemental 2). Boolean operators “AND” and “OR” were used to relate information between groups and according to each block of information, respectively. The groups of descriptors used in the systematic search for information were the following: (1) First block (outcome): (“blood pressure”) OR (“hypertension” [MeSH Terms]) OR (“high blood pressure”) OR (“systemic arterial hypertension”) OR (“systolic blood pressure”) OR (“diastolic blood pressure”). (2) Second block (exposure—anthropometric indicators of obesity): (“body mass ratio and squared height”) OR (“body mass index” [MeSH Terms]) OR (“bmi”) OR (“nutritional status”) OR (“overweight”) OR (“obesity” [MeSH Terms]) OR (“body fatness”) OR (“body composition” [MeSH Terms]) OR (“body fat”) OR (“Quetelet index” [MeSH Terms]) OR (“body roundness index”) OR (“body shape index”) OR (“waist/height ratio”) OR (“abdominal Obesity”) OR (“waist/hip ratio”) OR (“abdominal obesity”) OR (“waist Circumference” [MeSH Terms]) OR (“waist”) OR (“conicity index”) OR (“c index”) OR (“body fat index”) OR (“bai”) OR (“fat percentage”) OR (“body fat percentage”) OR (“triceps skinfold”) OR (“triceps skinfold thickness”) OR (“subscapular skinfold”) OR (“subscapular skinfold thickness”) OR (“suprailiac skinfold”) OR (“suprailiac fold”) OR (“suprailiac skinfold thickness”) OR (“skinfold iliac crest”) OR (“calf skinfold”) OR (“calffold”) OR (“skinfold thickness calf”) OR (“anthropometric indicators of obesity”) OR (“anthropometric indicators”) OR (“anthropometric indicators of body fat”). (3) Third block (population of interest): (“teenagers”) OR (“youth”) OR (“adolescence” [MeSH Terms]) OR (“schoolchildren”) OR (“students” [MeSH Terms]).

### Data extraction and Quality assessment

The information extracted from each study was as follows: author and year of publication, study location, sample size, age group, study design, anthropometric measures and indicators, instrument used/means of measuring blood pressure (BP), BP measurement recommendations, cutoff points estimated by studies, classification adopted for the identified BP values, indicators and diagnostic information for the predicted cutoff points (area under the curve (AUC)), sensitivity, specificity, positive predictive values (PPV), negative predictive values (NPV), optimal point criteria, measures of association for predicted results. Additionally, adjusted results were extracted from included studies and, when available, were presented according to sex and age group.

Although the use of cutoff points with AUC values lower than 0.70 (Akobeng, 2007; Fischer, Bachman & Jaeschke, 2003) is not recommended, studies included in this review that reported results that anthropometric obesity indicators had good predictive capacity for HBP used cutoff points with AUC < 0.70 to support such information. For this reason, in the present study, AUCs > 0.50 to < 0.70 were considered to have low diagnostic accuracy, AUCs ≥ 0.70 to < 0.90 were considered to have moderate predictive capacity, and AUCs ≥ 0.90 were considered to have high predictive capacity for the analyzed outcome (Akobeng, 2007; Fischer, Bachman & Jaeschke, 2003).

The assessment of the methodological quality/risk of bias of studies was carried out independently by two reviewers (LLB & TRL) using the QUADAS-2 methodological quality assessment tool (Whiting et al., 2010), which aims to assess the methodological quality/risk of bias of primary diagnostic accuracy studies. The tool consists of four main domains: (1) patient selection, (2) index test, (3) reference standard, and (4) patient flow through the study/time of the index test(s) and reference standard (“flow and time”). The assessment of each study is completed in four phases, and each domain is assessed in relation to the risk of bias  (Whiting et al., 2010) (Supplemental 3). The first three domains (patient selection, index test, and reference standard, respectively) are also evaluated with respect to applicability concerns. To help decision making regarding the risks of bias, the instrument has flagging questions. The flagging questions are answered as follows: “yes” (low risk of bias/high methodological quality), “no” (high risk of bias/low methodological quality) and “unclear” (insufficient information to allow for a judgment). If all flagging questions for a given domain are answered “yes”, the article’s risk of bias is considered “low”, whereas if any question is answered “no”, the article is considered to have some potential risk of bias. The answer “unclear” is only assigned to the item evaluated when there is not enough information to make a judgment. Also in relation to the assessment, although the risk of bias/methodological quality analysis tool does not have flagging questions for attributing judgment regarding the applicability of the strategy adopted by the study under analysis, the authors are requested to record information upon which the applicability judgment is performed and then classify the reason why the study met or did not meet such criteria (Whiting et al., 2010).

This instrument for assessing the risk of bias/methodological quality does not use a “quality score” classification, since the analysis of studies occurs in a segmented manner (according to items previously described). However, if a study was judged to be “low” in one or more domains related to bias or applicability, then it is appropriate to have an overall judgment of “low risk of bias” or “little concern about applicability” for that study. If a study was considered “high” or “not clear” in one or more domains, it could be considered “at risk of bias” or “with concerns about applicability”  (Whiting, Harbord & Kleijnen, 2005).

## Results

**Figure 1:**
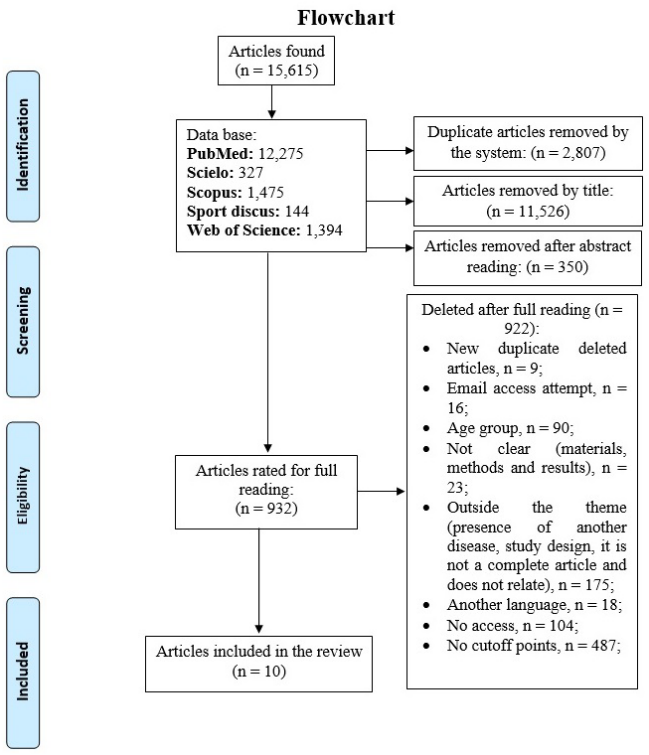
Flowchart of the systematic search for studies whose objective was to propose cutoff points for anthropometric indicators of obesity to predict HBP in adolescents.

Searches conducted in the investigated databases identified 15,615 studies, which after exclusion of duplicates, reading titles and abstracts, totaled 932 studies, whose information was analyzed in full. Of this total, 10 studies were part of the evidence summarization conducted in the present review (Fig. 1).

The evidence included in this review came from cross-sectional studies, four of them being carried out in the American continent (Beck, Da Silva Lopes & Pitanga, 2011; De Moraes & Da Veiga, 2014; Vogt Cureau & Reichert, 2013; Taylor & Hergenroeder, 2011), four in Asia (Abbaszadeh et al., 2017; Al-Bachir & Bakir, 2017; Febriana, Nurani & Julia, 2015; Kajale et al., 2014), one study in the African continent (Kruger et al., 2013) and one in Europe (Mazicioglu et al., 2010). All studies investigated the probable relationship of BMI in predicting high blood pressure levels. In addition to BMI, other anthropometric indicators of obesity such as WC (Beck, Da Silva Lopes & Pitanga, 2011; De Moraes & Da Veiga, 2014; Vogt Cureau & Reichert, 2013; Taylor & Hergenroeder, 2011; Abbaszadeh et al., 2017; Kajale et al., 2014; Mazicioglu et al., 2010), HC (Vogt Cureau & Reichert, 2013; Abbaszadeh et al., 2017), relaxed arm perimeter (RAP) (Mazicioglu et al., 2010), waist-to-hip ratio (WHR) (Abbaszadeh et al., 2017), WHtR (Beck, Da Silva Lopes & Pitanga, 2011; De Moraes & Da Veiga, 2014; Vogt Cureau & Reichert, 2013; Taylor & Hergenroeder, 2011; Abbaszadeh et al., 2017; Kajale et al., 2014; Mazicioglu et al., 2010), body adiposity index (BAI) (Vogt Cureau & Reichert, 2013) and conicity index (c index) (Beck, Da Silva Lopes & Pitanga, 2011) were investigated. Additionally, triceps and subscapularis skinfolds and the sum of skinfolds were used to calculate body fat percentage (%F) and the possible predictive capacity for HBP (Kajale et al., 2014; Mazicioglu et al., 2010) (Table 1).

**Table 1:** Characteristics of included studies.

Author	Country	Sample size (% female)	Age (years)	Study design	Anthropometric measures and indicators	BP measurement instrument	Recommendations for measuring BP
Al-Bachir & Bakir (2017)	Syria	2,064 (0.0)	18–19	Cross-sectional	Weight, height and BMI	Mercury sphygmomanometer	-Remain at rest before initial measurement;
Abbaszadeh et al. (2017)	Iran	1,046 (100.0)	11–19	Cross-sectional	Weight, height, BMI, WC, HC, WHR and WHtR	Mercury manometer	-Do not perform exercises before the measurements;
							-Remain at rest for 5 min before the initial measurement;
							-Do not eat chocolate, tea, coffee or heavy food before measuring;
							-One to three measurements on the right arm;
							-The average of the 3 measurements was considered;
							-Measurements were performed on different visits.
							(Suggested protocol (Roccella, 1996))
Febriana, Nurani & Julia (2015)	Indonesia	928 (47.2)	11–16	Cross-sectional	Weight, height, BMI, WC and WHtR	Oscillometric	-Remain at rest for 10 min before the initial measurement;
							-Sit in a comfortable position;
							-3 measurements on the right arm;
							-Measurements performed on different days;
							-5 min interval between measurements.
							(Suggested protocol (Roccella, 1996))
De Moraes & Da Veiga (2014)	Brazil	573 (68.3)	12–19	Cross-sectional	Weight, height, BMI, WC	Digital pressure gauge	-Remain at rest for 5 min before the first measurement;
							-Adolescents sitting;
							-2 measurements on the right arm;
							-Maximum difference between the two measurements was 5 mmHg;
							-The average of two measurements were considered;
							(Suggested protocol (Brandão et al., 2010))
Kajale et al. (2014)	China	6,380 (45.1)	6–18	Cross-sectional	Weight, height, BMI, WC, WHtR and sum of skinfolds	Mercury sphygmomanometer	-Remain 5 min at rest and seated;
							-The measurements were performed by pediatricians;
							-One to two measurements and confirmed by another evaluator;
							-10-minute interval between measurements;
							(Suggested protocol (Roccella, 1996))
Vogt Cureau & Reichert (2013)	Brazil	1,072 (54.2)	14–19	Cross-sectional	Weight, height, BMI, WC, HC, WHtR and BAI	Onrom digital device	-Remain at rest for 5 min before the initial measurement;
							-Do not practice physical activities in the hour before the measurement;
							-Do not consume alcohol, cigarettes or coffee up to 30 min before;
							-Empty the bladder before measuring;
							-Interval of 5 min between measurements;
							-3 measurements on the right arm;
							-The average of the two closest checks were considered;
							(Suggested protocol (Brandão et al., 2010))
Kruger et al. (2013)	South Africa	178 (61.2)	14–18	Cross-sectional	Weight, height, BMI, WC and WHtR	Onrom digital device	-Remain 5 min at rest and sitting, before the initial measurement;
							-2 measurements were performed on the right arm;
Taylor & Hergenroeder (2011)	USA	2,003 (52.1)	12–19	Cross-sectional	Weight, height, BMI and WC	U	-U;
Beck, Da Silva Lopes & Pitanga (2011)	Brazil	660 (51.9)	14–19	Cross-sectional	Weight, height, BMI, WC, WHtR and C index	Mercury sphygmomanometer auscultatory	-Remain 5 min at rest and sitting before the initial measurement;
							-One to three measurements on the right arm, the last measurement being considered;
							(Suggested protocol (Giuliano et al., 2005)).
Mazicioglu et al. (2010)	Turkey	2,860 (51.6)	11–17	Cross-sectional	Weight, height, BMI, WC, MUAC, TSF and %F	Aneroid sphygmomanometer	-Remain at rest and seated before the initial measurement;
							-2 measurements on the right arm, being considered the average of the two measurements;
							-Interval of 5 to 10 min between measurements;
							(Suggested protocol (Roccella, 1996))

**Notes.**

BP blood pressure BMI body mass index WC waist circumference WHtR waist-to-height-ratio WHR waist-hip-ratio HC hip circumference MUAC mid-upper-arm circumference C index conicity index BAI body adiposity index %F fat percentage TSF triceps skinfold thickness U uninformed suggested protocol (Roccella, 1996) Roccella (1996) suggested protocol (Brandão et al., 2010) Brandão et al. (2010) suggested protocol (Giuliano et al., 2005) Giuliano et al. (2005)

Eight studies used the 90th or 95th SBP and/or DBP percentiles to classify individuals with HBP, according to sex and age (Beck, Da Silva Lopes & Pitanga, 2011; De Moraes & Da Veiga, 2014; Vogt Cureau & Reichert, 2013; Taylor & Hergenroeder, 2011; Febriana, Nurani & Julia, 2015; Kajale et al., 2014; Kruger et al., 2013; Mazicioglu et al., 2010). One study followed the guidelines (Roccella, 1996) for classifying pressure levels determined according to age, sex and adjusted height (Abbaszadeh et al., 2017). Thus, in the aforementioned study (Abbaszadeh et al., 2017), if the mean of three measurements of SBP or DBP was greater than the 90th percentile and less than the 95th percentile, adolescents were classified as prehypertensive, if the mean of SBP or DBP was greater than the 95th percentile, adolescents were classified as hypertensive, and if the mean of the three measurements of SBP or DBP was less than the 90th percentile, adolescents were classified as normal blood pressure. In addition, adolescents with SBP > 120 mmHg and DBP > 80 mmHg, but with a percentile < 95, were considered prehypertensive (Abbaszadeh et al., 2017). In another study (Al-Bachir & Bakir, 2017), values of SBP > 135 mmHg and DBP > 89 mmHg were adopted to classify adolescents with HBP. Furthermore, eight studies indicated that anthropometric indicators of obesity had an acceptable predictive capacity to identify HBP in adolescents (Beck, Da Silva Lopes & Pitanga, 2011; Vogt Cureau & Reichert, 2013; Taylor & Hergenroeder, 2011; Abbaszadeh et al., 2017; Al-Bachir & Bakir, 2017; Febriana, Nurani & Julia, 2015; Kajale et al., 2014; Mazicioglu et al., 2010). However, only in one study (Beck, Da Silva Lopes & Pitanga, 2011) the cutoff points of the anthropometric indicators showed higher AUC values: BMI [AUC: 0.95 (95% CI [0.87–1.00])], WC [AUC: 0.96 (95% CI [0.92–1.00])] and WHtR [AUC: 0.93 (95% CI: 0.85-100)] among females; and WC [AUC: 0.80 (95% CI [0.72–0.89])] among males (Beck, Da Silva Lopes & Pitanga, 2011) (Table 2).

**Table 2:** Specific characteristics of included studies.

Author	Classification for hypertension	Identified cutoff	AUC (95% CI)	Sensitivity (95% CI)	Specificity (95% CI)	PPV (95% CI)	NPV (95% CI)	Optimum point criterion	Association measure for the identified cutoff point	Results
Al-Bachir & Bakir (2017)	SBP >135 mmHg and DBP >89 mmHg	BMI: 22.85 kg/m^2^ (SBP) 23.45 kg/m^2^ (DBP)	SBP: 0.52^a^ DBP: 0.59^a^	SBP: 0.45^a^ DBP: 0.50^a^	SBP: 0.59^a^ DBP: 0.67^a^	U	U	No	U	BMI was a good predictor of HBP.
Abbaszadeh et al. (2017)	SBP and/or DBP > 120/80 mmHg or >90th percentile according to sex, age and height	WC: 78.50 cm (SBP); 79.50 cm (DBP); BMI: 23.40 kg/m^2^ (SBP); 24.30 kg/m^2^ (DBP); WHtR: 0.48 (SBP); 0.48 (DBP); WHR: 0.79 (SBP); 0.79 (DBP);	♀ : SBP: BMI: 0.71 (0.66–0.76) WC: 0.73 (0.68–0.78) WHR: 0.62 (0.57–0.67) WHtR: 0.73 (0.67–0.77) DBP: BMI: 0.67 (0.62–0.73) WC: 0.71 (0.67–0.76) WHR: 0.62 (0.57–0.67) WHtR: 0.72 (0.63–0.77)	SBP: BMI: 0.59 (0.50–0.68) WC: 0.62 (0.54–0.70) WHR: 0.70 (0.62–0.78) WHtR: 0.71 (0.63–0.79) DBP: BMI: 0.50 (0.41–0.59) WC: 0.58 (0.49–0.67) WHR: 0.71 (0.63–0.79) WHtR: 0.71 (0.63–0.79)	SBP: BMI: 0.74 (0.71–0.77) WC: 0.73 (0.70–0.76) WHR: 0.51 (0.48–0.54) WHtR: 0.66 (0.62–0.70) DBP: BMI: 0.80 (0.77–0.83) WC: 0.75 (0.72–0.78) WHR: 0.59 (0.56–0.62) WHtR: 0.67 (0.64–0.70)	SBP: BMI: 0.24 (0.19–0.29) WC: 0.24 (0.19–0.29) WHR: 0.16 (0.13–0.19) WHtR: 0.22 (0.18–0.27) DBP: BMI: 0.23 (0.18–0.28) WC: 0.23 (0.18–0.28) WHR: 0.15 (0.12–0.18) WHtR: 0.21 (0.17–0.25)	SBP: BMI: 0.93 (0.91–0.95) WC: 0.93 (0.91–0.95) WHR: 0.93 (0.91–0.95) WHtR: 0.94 (0.92–0.96) DBP: BMI: 0.93 (0.91–0.95) WC: 0.94 (0.92–0.96) WHR: 0.93 (0.91–0.95) WHtR: 0.95 (0.93–0.97)	Youden index	U	The study demonstrated that WHtR is the best anthropometric indicator for the determination of HBP, compared to WC, BMI and WHR in the present study.
Febriana, Nurani & Julia (2015)	percentile BP > 90th	♀ and ♂ : WHtR: 0.45 BMIZ: 0.51	U	♀ : SBP: WHtR: 0.72 (0.65–0.78) BMIZ: 0.83 (0.78–0.89) DBP: WHtR: 0.71 (0.60–0.74) BMIZ: 0.81 (0.75–0.87) ♂ : SBP: WHtR: 0.72 (0.65–0.78) BMIZ: 0.81 (0.77–0.86) DBP: WHtR: 0.76 (0.67–0.78) BMIZ: 0.82 (0.77–0.87)	♀ : SBP: WHtR: 0.73 (0.68–.,79) BMIZ: 0.75 (0.70–0.80) DBP: WHtR: 0.72 (0.64–0.73) BMIZ: 0.73 (0.66–0.77) ♂ : SBP: WHtR: 0.77 (0.72–0.83) BMIZ: 0.77 (0.72–0.83) DBP: WHtR: 0.73 (0.67–0.78) BMIZ: 0.72 (0.67–0.77)	U	U	No	U	The results of the study demonstrate that the cutoff points for BMIZ and WHtR were adequate and good predictors of HBP.
De Moraes & Da Veiga (2014)	SBP and DBP > 120/80 mmHg or >90th percentile according to sex, age and height	♀ : 67.70 cm ♂ : 71.50 cm	♀ : WC: 0.70 (0.62–0.78) ♂ : WC: 0.61 (0.54–0.69)	♀ : WC: 0.65^a^ ♂ : WC: 0.60^a^	♀ : WC: 0.64 ♂ : WC: 0.60	U	U	No	U	WC did not show good accuracy in identifying HBP in both sexes.
Kajale et al. (2014)	SBP and/or DBP percentil >90th e <95th—pre hypertension and SBP/DBP >95th—hypertension	♀ : WC: 77.00 cm (10–14 years); 87.00 cm (15–18 years); BMI: 19.20 kg/m^2^ (10–14 years); 22.70 kg/m^2^ (15–18 years); WHtR: 0.51 (10–14 years); 0.55 (15–18 years); TSF: 16.80 mm (10–14 years); 18.40 mm (15–18 years); WP: 13.50 cm (10–14 years); 13.70 cm (15–18 years). ♂ : WC: 81.00 cm (12–15 years); 90.00 cm (16–18 years); BMI: 21.80 kg/m^2^ (12–15 years); 26.10 kg/m^2^ (16–18 years); WHtR: 0.53 (12–15 years); 0.53 (16–18 years); TSF: 14.50 mm (12–15 years); 17.4 mm (16–18 years); WP: 14.80 cm (12–15 years); 15.90 cm (16–18 years).	U	Index variation from 60 to 90%	Index variation from 60 to 90%	U	U	No	Multiple logistic regression^**^	The five anthropometric measures (WC, BMI, WHtR, TSF and WP) were associated with HBP, showing a good ability to predict hypertension.
Vogt Cureau & Reichert (2013)	Percentile >95 considered with HBP for adolescents; >140/90 mmHg for adults.	♀ : WC: 71.26 cm BMI: 22.15 kg/m^2^ WHtR: 0.45 BAI: 28.78 ♂ : WC: 75.64 cm; BMI: 22.23 kg/m^2^; WHtR: 0.44; BAI: 23.64;	♀ : WC: 0.71 (0.67–0.74) BMI: 0.71 (0.67–0.74) WHtR: 0.73 (0.69–0.77) BAI: 0.71 (0.67–0.75) ♂ : WC: 0.63 (0.58–0.67) BMI: 0.64 (0.60–0.68) WHtR: 0.63 (0.59–0.68) BAI: 0.63 (0.58–0.67)	♀ : WC: 0.64 (0.53–0.74) BMI: 0.67 (0.56–0.76) WHtR: 0.69 (0.58–0.78) BAI: 0.66 (0.56–0.76) ♂ : WC: 0.59 (0.51–0.67) BMI: 0.60 (0.52–0.67) WHtR: 0.61 (0.53–0.68) BAI: 0.58 (0.50–0.66)	♀ : WC: 0.65 (0.60–0.69) BMI: 0.66 (0.62–0.70) WHtR: 0.69 (0.64–0.73) BAI: 0.67 (0.62–0.71) ♂ : WC: 0.59 (0.53–0.64) BMI: 0.60 (0.55–0.65) WHtR: 0.61 (0.56–0.67) BAI: 0.58 (0.53–0.64)	U	U	No	Poisson regression ♀ : WC: 2.79 (1.68–4.64) BMI: 3.05 (1.85–5.02) WHtR: 3.88 (2.29–6.59) BAI: 3.65 (2.19–6.09) ♂ : WC: 1.64 (1.27–2.11) BMI: 1.73 (1.34–2.24) WHtR: 1.81 (1.39–2.34) BAI: 1.55 (1.20–1.99)	The results indicated a good association of anthropometric indicators of obesity with HBP, in addition, WHtR proved to be more effective in predicting HBP in adolescents.
Kruger et al. (2013)	SBP/DBP >90th percentile for age and sex	WHtR: 0.41	WHtR: 0.56^a^	WHtR: 0.64^a^	WHtR: 0.50^a^	WHtR: 0.56^a^	WHtR: 0.54^a^	No	Logistic regression 2.35 (0.96–5.75)	The cutoff value was lower than that proposed in the literature, showing weak correlation and predictive power to identify HBP.
Taylor & Hergenroeder (2011)	SBP/DBP >90th percentile for age and sex	♀ : WC: 81.00 cm ♂ : WC: 80.50 cm	♀ : WC: 0.65^a^ ♂ : WC: 0.77^a^	♀ : WC: 0.57^a^ ♂ : WC: 0.74^a^	♀ : WC: 0.74^a^ ♂ : WC: 0.71^a^	U	U	No	Logistic regression ♀ : Excess weight and WC >cutoff point: 9.05 (1.44–56.83) ♂ : Normal weight and WC >cutoff point: 4.06 (1.64–10.05) Excess weight and WC >cutoff point: 5.24 (1.48–18.6)	Values above the cutoff points were shown to be good predictors to identify individuals with HBP.
Beck, Da Silva Lopes & Pitanga (2011)	SBP/DBP percentile >90 and <95—pre-hypertension and SBP/DBP Percentile >95 and <99—hypertension	♀ : BMI: 24.00 kg/m^2^ WC: 82.40 cm WHtR: 0.48 C index: 1.14 ♂ : BMI: 21.90 kg/m^2^; WC: 75.40 cm; WHtR: 0.43; C index: 1.13;	♀ : BMI: 0.95 (0.87–1.00) WC: 0.96 (0.92–1.00) WHtR: 0.93 (0.85–1.00) C index: 0.74 (0.50–0.98) ♂ : BMI: 0.79 (0.68–0.89) WC: 0.80 (0.72–0.89) WHtR: 0.77 (0.66–0.88) C index: 0.69 (0.56–0.81)	♀ : BMI: 1.00 WC: 1.00 WHtR: 1.00^a^ C index: 0.75^a^ ♂ : BMI: 0.72^a^ WC: 0.78^a^ WHtR: 0.72^a^ C index: 0.67^a^	♀ : BMI: 0.84^a^ WC: 0.92^a^ WHtR: 0.85^a^ C index: 0.67^a^ ♂ : BMI: 0.68^a^ WC: 0.67^a^ WHtR: 0.67^a^ C index: 0.57^a^	U	U	No	U	BMI, WC and WHtR were good predictors of HBP in both sexes.
Mazicioglu et al. (2010)	SBP and/or DBP percentile >95	♀ 11–14 years: Weight: >56.80 kg Height: >157.50 cm BMI: >20.56 kg/m^2^ WC: >65.50 cm WHtR: >0.40 TSF: >20.40 mm WASR: >0.42 MUAC: >22.50 cm AFA: >13.24 cm^2^ %F: >41.90 AS: >161.80 ♀ 15–17 years: Weight: >58.10 kg Height: >164.20 cm BMI: >23.14 kg/m^2^ WC: >70.60 cm WHtR: >0.41 TSF: >16.80 mm WASR: >0.51 MUAC: >24.90 cm AFA: >15.67 cm^2^ %F: >41.43 AS: >161.00 ♂ 11–14 years: Weight: >47.00 kg Height: >152.50 cm BMI: >20.12 kg/m^2^ WC: >65.40 cm WHtR: >0.42 TSF: >10.00 mm WASR: >0.41 MUAC: >21.40 cm AFA: >10.49 cm^2^ %F: >30.79 AS: >156.50 ♂ 15–17 years: Weight: >65.70 kg Height: >152.50 cm BMI: >22.10 kg/m^2^ WC: >78.40 cm WHtR: >0.46 TSF: >10.10 mm WASR: >0.43 MUAC: >24.60 cm AFA: >10.49 cm^2^ %F: >24.71 AS: >174.00	♀ 11–14 years: Weight: 0.64 (0.60–0.67) Height.: 0.52 (0.48–0.55) BMI: 0.65 (0.62–0.69) WC: 0.68 (0.64–0.71) WHtR: 0.68 (0.65–0.72) TSF: 0.61 (0.57–0.64) WASR: 0.68 (0.64–0.71) MUAC: 0.64 (0.60–0.67) AFA: 0.62 (0.59–0.66) %F: 0.58 (0.54–0.61) AS: 0.52 (0.48–0.56)	U	U	U	U	No	Logistic regression ♀ : SBP: Height.: 0.12 (0.02–0.22) BMI: 0.75 (0.54–0.96) WC: 0.33 (0.24–0.43) WHtR: 48.26 (32.27–64.25) TSF: 0.25 (0.14–0.37) WASR: 45.25 (28.99–61.51) MUAC; 0.57 (0.31–0.83) AFA: 0.32 (0.20–0.43) %F: 0.10 (0.04–0.17) AS: 0.12 (0.04–0.21) Age: 0.05 (−0.35–0.45) DBP: Height.: 0.09 (0.01–0.16) BMI: 0.49 (0.33–0.65) WC: 0.25 (0.17–0.32) WHtR: 36.27 (24.17–48.42) TSF: 0.22 (0.13–0.31) WASR: 30.44 (18.08–42.80) MUAC: 0.58 (0.38–0.77) AFA: 0.23 (0.15–0.31) %F: 0.09 (0.04–0.14) AS: 0.13 (0.06–0.19) Age: 0.46 (0.16–0.76) ♂ : SBP: Height.: 0.14 (0.08–0.20) BMI: 0.88 (0.67–1.10) WC: 0.33 (0.26–0.41) WHtR: 47.17 (33.04–61.30) TSF: 0.29 (0.15–0.43) WASR: 47.71 (33.34–62.08) MUAC: 0.80 (0.59–1.02) AFA: 0.32 (0.20–0.43) %F: 0.08 (0.01–0.16) AS: 0.12 (0.06–0.17)	WC and BMI were good predictors of SBP and DBP in the >95th percentile.
		15–17 years: Weight: 0.62 (0.58–0.65) Height.: 0.52 (0.49–0.56) BMI: 0.63 (0.59–0.66) WC: 0.59 (0.56–0.63) WHtR: 0.59 (0.55–0.63) TSF: 0.56 (0.52–0.59) WASR: 0.55 (0.52–0.59) MUAC: 0.53 (0.49–0.56) AFA: 0.56 (0.52–0.60) %F: 0.55 (0.51–0.58) AS: 0.60 (0.55–0.63) ♂ : 11–14 years: Weight: 0.71 (0.67–0.75) Height.: 0.60 (0.55–0.64) BMI: 0.69 (0.65–0.73) WC: 0.74 (0.70–0.76) WHtR: 0.68 (0.64–0.71) TSF: 0.59 (0.55–0.63) WASR: 0.70 (0.67–0.74) MUAC: 0.75 (0.71–0.78) AFA: 0.64 (0.60–0.68) %F: 0.52 (0.48–0.56) AS: 0.58 (0.54–0.62) 15–17 years: Weight: 0.62 (0.59–0.66) Height.: 0.51 (0.47–0.54) BMI: 0.64 (0.60–0.67) WC: 0.65 (0.62–0.70) WHtR: 0.66 (0.62–0.70) TSF: 0.60 (0.56–0.63) WASR: 0.70 (0.67–0.74) MUAC: 0.62 (0.71–0.78) AFA: 0.60 (0.60–0.68) %G: 0.57 (0.54–0.61) AS: 0.50 (0.46–0.53)						Age: 0.53 (0.13–0.93) DBP: Height.: 0.09 (0.04–0.14) BMI: 0.55 (0.38–0.72) WC: 0.22 (0.16–0.28) WHtR: 31.23 (20.26–42.19) TSF: 0.25 (0.14–0.36) WASR: 32.03 (20.89–43.18) MUAC: 0.56 (0.39–0.72) AFA: 0.25 (0.16–0.34) %F: 0.013 (0.08–0.03) AS: 0.08 (0.03–0.12) Age: 0.32 (0.01–0.63)	

**Notes.**

HBP high blood pressure SBP systolic blood pressure DBP diastolic blood pressure BM body mass BMI body mass index WC waist circumference WHtR waist-to-height-ratio WHR waist-hip-ratio HC hip circumference WP wrist perimeter C index conicity index BAI body adiposity index %F fat percentage TSF tríceps skinfold thickness BMIZ body mass index *z* score WASR waist-to-arm-span ratio MUAC mid-upper-arm circumference AFA arm-fat area AS arm span ♀ girls ♂ boys AUC area under the curve 95% CI confidence interval 95% PPV positive predictive values NPV negative predictive values U uninformed Sens. sensitivity Spec. specificity

a The study did not report the confidence interval.

b Odds ratio not reported in the study.

Seven studies proposed cutoff points for WC (Beck, Da Silva Lopes & Pitanga, 2011; De Moraes & Da Veiga, 2014; Vogt Cureau & Reichert, 2013; Taylor & Hergenroeder, 2011; Abbaszadeh et al., 2017; Kajale et al., 2014; Mazicioglu et al., 2010) and seven studies proposed cutoff points for BMI (Beck, Da Silva Lopes & Pitanga, 2011; Vogt Cureau & Reichert, 2013; Abbaszadeh et al., 2017; Al-Bachir & Bakir, 2017; Febriana, Nurani & Julia, 2015; Kajale et al., 2014; Mazicioglu et al., 2010). Additionally, cutoff points were developed for WHtR (Beck, Da Silva Lopes & Pitanga, 2011; Vogt Cureau & Reichert, 2013; Abbaszadeh et al., 2017; Febriana, Nurani & Julia, 2015; Kajale et al., 2014; Kruger et al., 2013; Mazicioglu et al., 2010), WHR (Abbaszadeh et al., 2017), BAI (Vogt Cureau & Reichert, 2013), triceps skinfold (TSF) (Kajale et al., 2014; Mazicioglu et al., 2010), wrist perimeter (WP) (Kajale et al., 2014), c index (Beck, Da Silva Lopes & Pitanga, 2011), body mass (BM), height, waist-to-arm-span ratio (WASR), mid-upper-arm circumference (MUAC), arm fat area (AFA), arm span (AS) and %F (Mazicioglu et al., 2010) (Table 2).

Four studies reported in detail the selection of subjects, with high methodological quality/low risk of bias (Beck, Da Silva Lopes & Pitanga, 2011; De Moraes & Da Veiga, 2014; Vogt Cureau & Reichert, 2013; Kajale et al., 2014). Seven studies showed low concern about the applicability of results (Beck, Da Silva Lopes & Pitanga, 2011; De Moraes & Da Veiga, 2014; Vogt Cureau & Reichert, 2013; Abbaszadeh et al., 2017; Al-Bachir & Bakir, 2017; Febriana, Nurani & Julia, 2015; Kajale et al., 2014) (Table 3). Regarding the index test, only two studies showed high methodological quality/low risk of bias with regard to the way in which anthropometric measurements were performed and the presentation of anthropometric indicators of obesity (Al-Bachir & Bakir, 2017; Kajale et al., 2014). Regarding the adopted reference standard, only one study showed high methodological quality/low risk of bias (Abbaszadeh et al., 2017) and two studies showed low concern about applicability of results (Abbaszadeh et al., 2017; Febriana, Nurani & Julia, 2015). In addition, the same studies performed BP measurements at different visits. With regard to domain four—flow and time, only two studies showed low risk of bias (Abbaszadeh et al., 2017; Febriana, Nurani & Julia, 2015) (Table 3).

**Table 3:** Assessment of the methodological quality/risk of bias of included studies.

Studies	Risk of bias				Applicability		
	Patient selection	Index test	Reference standard	Flow and time	Patient selection	Index test	Reference standard
Al-Bachir & Bakir (2017)	Unclear	Low	Unclear	High	Low	Low	Unclear
Abbaszadeh et al. (2017)	High	High	Low	Low	Low	Low	Low
Febriana, Nurani & Julia (2015)	High	High	High	Low	Low	High	Low
De Moraes & Da Veiga (2014)	Low	High	High	High	Low	Low	High
Kajale et al. (2014)	Low	Low	High	High	Low	Low	High
Vogt Cureau & Reichert (2013)	Low	High	High	High	Low	Low	High
Kruger et al. (2013)	High	High	High	High	High	Unclear	High
Taylor & Hergenroeder (2011)	High	High	Unclear	Unclear	High	High	Unclear
Beck, Da Silva Lopes & Pitanga (2011)	Low	High	High	High	Low	Low	High
Mazicioglu et al. (2010)	High	High	High	High	High	High	High

## Discussion

This systematic review study synthesized evidence from ten cross-sectional studies, which involved a total of 17,764 participants. The information summarized in this review indicated that anthropometric indicators (BMI, WC, WHtR, TSF, BAI, C Index, BM, WASR, AFA, MUAC, FP and AS) had low diagnostic accuracy for HBP screening in adolescents.

In seven studies (Beck, Da Silva Lopes & Pitanga, 2011; Vogt Cureau & Reichert, 2013; Abbaszadeh et al., 2017; Al-Bachir & Bakir, 2017; Febriana, Nurani & Julia, 2015; Kajale et al., 2014; Mazicioglu et al., 2010), the cutoff points for BMI to be used in screening for HBP in adolescents showed low predictive capacity (AUC < 0.70). In the study carried out in the South of Brazil with 660 adolescents of both sexes, the AUC values for BMI were 0.79 (95% CI [0.68–0.89]) for male adolescents (Beck, Da Silva Lopes & Pitanga, 2011). In another study carried out in the South of Brazil with 1,702 adolescents of both sexes, the AUC values identified for BMI were 0.71 (95% CI [0.67–0.74]) for female adolescents and 0.64 (95% CI [0.60–0.68]) for male adolescents (Vogt Cureau & Reichert, 2013). In one study carried out in Iran with 1,046 female adolescents, the AUC values obtained using BMI as the investigated anthropometric obesity indicator were 0.71 (95% CI [0.66–0.76]) for SBP, and 0.67 (95% CI [0.62–0.73]) for DBP (Abbaszadeh et al., 2017). A study carried out in Syria with 2,064 male adolescents showed AUC value for SBP of 0.52, while for DBP, the AUC value identified was 0.59 (Al-Bachir & Bakir, 2017). It is noteworthy that the 95% CI values for the described AUC were not available (Al-Bachir & Bakir, 2017). In another study (Mazicioglu et al., 2010), the AUC values identified for BMI as a possible tool to be used in the screening for HBP in female adolescents aged 11-14 years were 0.65 (95% CI [0.62–0.69]), while for female adolescents aged 15-17 years, the AUC values verified were 0.63 (95% CI [0.59–0.66]) (Mazicioglu et al., 2010). In addition, for male adolescents aged 11-14 years, the AUC values verified were 0.69 (95% CI [0.65–0.73]), while for male adolescents aged 15-17 years, the identified AUC values were 0.64 (95% CI [0.60–0.67]) (Mazicioglu et al., 2010). It is noteworthy that although two studies (Febriana, Nurani & Julia, 2015; Kajale et al., 2014) did not present AUC values for the proposed cutoff points, the authors concluded that the cutoff points had good diagnostic capacity for HBP. When stratified by sex, one study (Beck, Da Silva Lopes & Pitanga, 2011) found that BMI had moderate predictive capacity to identify HBP in female adolescents (AUC: 0.95; 95% CI [0.87–1.00]). In these studies, the proposed cutoff points ranged from 19.2 kg/m^2^ to 24.0 kg/m^2^ for females and from 21.8 kg/m^2^ to 26.1 kg/m^2^ for males (Beck, Da Silva Lopes & Pitanga, 2011; Kajale et al., 2014). One study presented the cutoff point as *z* score (Febriana, Nurani & Julia, 2015). This difference in cutoff points may be due to the different age groups of studies analyzed, which results in different body mass (BM) (in Kgs or z-scores) and height values.

Most studies (5/7 studies) whose objective was to propose cutoff points for WC in order to identify HBP reported low predictive capacity of this anthropometric obesity indicator (95% CI of AUC > 0.50 to < 0.70). However, one study (Beck, Da Silva Lopes & Pitanga, 2011) showed AUC values > 0.70, indicating high diagnostic test accuracy (Akobeng, 2007; Fischer, Bachman & Jaeschke, 2003) for female adolescents (AUC 0.96; 95% CI [0.92–1.00]) and moderate diagnostic test accuracy (Akobeng, 2007; Fischer, Bachman & Jaeschke, 2003) for male adolescents (AUC 0.80; 95% CI [0.72–0.89]). Similarly, another study (Mazicioglu et al., 2010) also identified that the thresholds verified for the investigated obesity indicator (WC) had moderate predictive capacity (Akobeng, 2007; Fischer, Bachman & Jaeschke, 2003) (AUC > 0.70) for male adolescents aged 11–14 years (AUC 0.74; 95% CI [0.70–0.76]). Regarding cutoff points proposed by these studies, WC differences of up to 16.8 cm among studies for females and up to 15.0 cm for males were described. It is possible that WC measurement protocols adopted by studies such as the midpoint between the upper edge of the iliac crest and the last rib (Beck, Da Silva Lopes & Pitanga, 2011) or the highest point of the iliac crest (Taylor & Hergenroeder, 2011) or for not showing how WC was measured (Mazicioglu et al., 2010) may have contributed to differences between proposed cutoff points. Another aspect that needs to be reported is the fact that the three studies (Beck, Da Silva Lopes & Pitanga, 2011; Taylor & Hergenroeder, 2011; Mazicioglu et al., 2010) that reported acceptable predictive capacity of WC had low methodological quality/high risk of bias.

In seven studies (Beck, Da Silva Lopes & Pitanga, 2011; Vogt Cureau & Reichert, 2013; Abbaszadeh et al., 2017; Febriana, Nurani & Julia, 2015; Kajale et al., 2014; Kruger et al., 2013; Mazicioglu et al., 2010) the cutoff points for WHtR showed low predictive capacity to identify HBP in adolescents. However, in a study conducted with the participation of 660 schoolchildren aged 14–19 years (Beck, Da Silva Lopes & Pitanga, 2011), the AUC value for WHtR to determine HBP in female adolescents was 0.93 (95% CI [0.85–1.00]), demonstrating moderate predictive capacity (Akobeng, 2007; Fischer, Bachman & Jaeschke, 2003). It is necessary to highlight that only one study (Abbaszadeh et al., 2017) used a large number of diagnostic strategies (AUC, sensitivity, specificity, PPV and NPV and the optimal point) to describe cutoff points. However, this study was carried out only with female adolescents. Regarding WHtR values to be used to identify adolescents with HBP, variations from 0.41 to 0.55 for females and from 0.42 to 0.53 for males were found (Beck, Da Silva Lopes & Pitanga, 2011; Vogt Cureau & Reichert, 2013; Abbaszadeh et al., 2017; Febriana, Nurani & Julia, 2015; Kajale et al., 2014; Kruger et al., 2013; Mazicioglu et al., 2010).

According to studies included in the present review, the anthropometric indicators TSF, BAI, C index, BM, WASR, AFA, MUAC, FP and AS showed low predictive capacity to identify HBP in adolescents (AUC > 0.50 to < 0.70). However, only one study  (Mazicioglu et al., 2010) showed AUC values greater than 0.70 for MUAC with AUC = 0.75 (95% CI [0.71–0.78]) for male adolescents aged 11–14 years, indicating that MUAC had moderate predictive capacity to identify HBP in adolescents (Akobeng, 2007; Fischer, Bachman & Jaeschke, 2003). It is speculated that AUC values < 0.70 may be related to the fact that although these anthropometric indicators are indeed used to discriminate obesity (whole body, localized or abdominal body fat), they may have limited ability to distinguish adolescents positive or not for HBP (Fischer, Bachman & Jaeschke, 2003). However, this assumption is restricted to results derived from studies in which information regarding this diagnostic measure was described (Beck, Da Silva Lopes & Pitanga, 2011; Vogt Cureau & Reichert, 2013; Abbaszadeh et al., 2017), since some of the studies added to the present review did not contain such information (Kajale et al., 2014; Mazicioglu et al., 2010).

This review identified high risk of bias with regard to subject selection and measurement procedures for anthropometric indicators of obesity in seven studies (Beck, Da Silva Lopes & Pitanga, 2011; De Moraes & Da Veiga, 2014; Vogt Cureau & Reichert, 2013; Taylor & Hergenroeder, 2011; Abbaszadeh et al., 2017; Kruger et al., 2013; Mazicioglu et al., 2010), and high risk of bias in the measurement of blood pressure levels in five studies (Beck, Da Silva Lopes & Pitanga, 2011; De Moraes & Da Veiga, 2014; Vogt Cureau & Reichert, 2013; Kruger et al., 2013; Mazicioglu et al., 2010). Furthermore, one study did not make it clear how procedures to measure blood pressure levels were performed (Taylor & Hergenroeder, 2011). Another aspect that deserves attention is that although eight studies (Beck, Da Silva Lopes & Pitanga, 2011; Vogt Cureau & Reichert, 2013; Taylor & Hergenroeder, 2011; Abbaszadeh et al., 2017; Al-Bachir & Bakir, 2017; Febriana, Nurani & Julia, 2015; Kajale et al., 2014; Kruger et al., 2013; Mazicioglu et al., 2010) have concluded that the anthropometric indicators of obesity had good predictive capacity for HBP, only in one study (Beck, Da Silva Lopes & Pitanga, 2011) the AUC values were acceptable for use in a clinical context (Akobeng, 2007; Fischer, Bachman & Jaeschke, 2003). Thus, in addition to high sensitivity and specificity values, the stipulated cut-off points must have high LR+ and low LR-values, in addition to allowing a definitive diagnostic condition for the investigated health outcome, regardless of the estimated prevalence. In this context, the use of diagnostic measures such as PPV and NPV are suggested.

Although the use of anthropometric indicators as diagnostic tools to be used for screening for HBP in adolescents has recognized clinical relevance, ethnicity can play a determining role in growth patterns, and consequently, in the precision and accuracy of anthropometric indicators used. Thus, not considering ethnic variation in the assessment of anthropometric obesity indexes to be used for screening for HBP in adolescents can lead to the identification of inaccurate results. However, even though the summarized evidence has been heterogeneous with respect to the investigated populations (adolescents from different regions of the globe—different ethnicities), the small number of studies impaired proposing results and possible suggestion of thresholds to be adopted for the diagnosis of HBP according to ethnicity.

This review presents strengths and limitations that must be considered. The large number of databases investigated (five) in order to identify evidence related to the topic of interest is considered a strong point of this review. Additionally, the analysis of studies in three different languages is another strong point of this review. Despite the careful search carried out by the researchers, it is possible that studies related to the theme covered in this review have not been identified, which is considered a limitation. Thus, the search in a greater number of databases and in the gray literature (for example, course conclusion works and specialization monographs) could contribute to the fact that possible information regarding the theme is not left out of the study. Moreover, the systematic search in other languages could also contribute to bringing new evidence on the investigated topic.

## Conclusion

Based on the summarized information, caution is suggested in the use of anthropometric indicators of obesity in the HBP screening in adolescents. Although there were studies that have suggested the use of these indicators, these suggestions were not based on AUC measures with high predictive capacity. Thus, further studies are needed that report a high predictive capacity of anthropometric indicators of obesity for the HBP screening.

##  Supplemental Information

10.7717/peerj.13590/supp-1 Supplemental Information 1 PRISMA checklist

10.7717/peerj.13590/supp-2 Supplemental Information 2 Descriptors and strategies used in the systematic search in the databases

10.7717/peerj.13590/supp-3 Supplemental Information 3 Guidance on the use of the methodological quality/risk of bias assessment instrument for studies of primary diagnostic accuracy (QUADAS-2)

## Additional Information and Declarations

### Data Availability

The following information was supplied regarding data availability: The raw data are available in the Supplementary Files.
