# Supplementary material for: Accuracy of anthropometric indicators of obesity to identify high blood pressure in adolescents—systematic review
Source: PeerJ. 2022 Aug 9;10:e13590. doi: 10.7717/peerj.13590 (PMC9373973; doi:10.7717/peerj.13590)
Supplement: Supplemental Information 2 [file peerj-10-13590-s002.docx]

**Supplemental 2**. Descriptors and strategies used in the systematic search in the investigated databases.

**First block - Outcome**

**English language terms:** “blood pressure”; “hypertension”; “high blood pressure”; “systemic arterial hypertension”; “systolic blood pressure”; “diastolic blood pressure”; “blood pressure”.

**Portuguese language terms:** “pressão arterial”; “hipertensão”; “pressão arterial alta”; “hipertensão arterial sistêmica”; “pressão arterial sistólica”; “pressão arterial diastólica”; “pressão arterial”.

**Spanish language terms:** “presión arterial”; “hipertensión”; “presión arterial alta”; “hipertensión arterial sistémica”; “presión arterial sistólica”; “presión arterial diastólica”; “presión arterial”.

**Second block - Exhibition**

**English language terms:** "body mass ratio and squared height"; "body mass index"; "body mass index"; “bmi”; "nutritional status"; “overweight”; “obesity”; “overweight”; "body fatness”; “body composition"; "body fat"; "Quetelet index"; "body roundness index"; "body shape index"; "waist/height ratio"; "waist/height ratio"; "abdominal obesity"; "waist-height ratio"; "waist-height ratio"; "waist/hip ratio"; "waist/hip ratio"; "abdominal obesity"; "waist circumference"; "waist circumference"; “waist”; "conicity index"; "c index"; "body fat index"; “bai”; "fat percentage"; "body fat percentage"; "triceps skinfold"; "triceps skinfold thickness"; "subscapular skinfold"; "subscapular skinfold thickness"; "suprailiac skinfold"; "suprailiac fold"; "suprailiac skinfold thickness"; "skinfold iliac crest"; "calf skinfold"; “calffold” "calf skinfold"; "skinfold thickness calf"; “anthropometric indicators of obesity"; "anthropometric indicators"; "anthropometric indicators of body fat".

**Portuguese language terms:** "razão de massa corporal e altura ao quadrado"; "índice de massa corporal"; "índice de massa corporal"; "IMC"; "estado nutricional"; "excesso de peso"; "obesidade"; "excesso de peso"; "gordura corporal"; “composição corporal"; "gordura corporal"; "índice de quetelet"; "índice de arredondamento corporal"; "índice de forma corporal"; "proporção cintura / estatura"; "proporção cintura / estatura"; "razão cintura / estatura"; "obesidade abdominal"; "razão cintura-estatura"; "proporção cintura-altura"; "proporção cintura / quadril"; "proporção cintura / quadril"; "obesidade abdominal"; "circunferência da cintura"; "circunferência da cintura"; "cintura"; "índice de conicidade"; "índice c"; "índice de gordura corporal"; "IAC"; "porcentagem de gordura"; "porcentagem de gordura corporal"; "dobra cutânea tríceps"; "espessura da dobra cutânea tríceps"; "dobra cutânea subescapular"; "espessura da dobra cutânea subescapular"; "dobra cutânea suprailíaca"; "prega suprailíaca"; "dobra cutânea suprailíaca"; "dobra cutânea ilíaca"; "espessura de dobra cutânea"; "indicadores antropométricos de obesidade"; "indicadores antropométricos"; "indicadores antropométricos de gordura corporal".

**Spanish language terms:** “relación de masa corporal y altura al cuadrado"; "índice de masa corporal"; "índice de masa corporal"; "IMC"; "estado nutricional"; "sobrepeso"; "obesidad"; "sobrepeso"; "grasa corporal"; "composición corporal"; "grasa corporal"; "índice de quetelet"; "índice de redondeo corporal"; "índice de forma corporal"; "relación cintura / altura"; "relación cintura / altura"; "relación cintura / altura"; "obesidad abdominal"; "relación cintura-altura"; "relación cintura-altura"; "proporción cintura cadera"; "proporción cintura cadera"; "obesidad abdominal"; "circunferencia de la cintura"; "circunferencia de la cintura"; "cintura"; "índice de ahusamiento"; "índice c"; "índice de grasa corporal"; "IAC"; "porcentaje de grasa"; "porcentaje de grasa corporal"; "pliegue cutáneo del tríceps"; "espesor del pliegue cutáneo del tríceps"; "pliegue cutáneo subescapular"; "espesor del pliegue cutáneo subescapular"; "pliegue cutáneo suprailíaco"; "pliegue suprailíaco"; "pliegue cutáneo suprailíaco"; "pliegue cutáneo ilíaco"; "espesor del pliegue cutáneo"; "indicadores antropométricos de obesidad"; "indicadores antropométricos"; "indicadores antropométricos de grasa corporal".

**Third block – Population of interest**

**English language terms:** "teenagers"; "youth"; "adolescence"; "schoolchildren"; "students".

**Portuguese language terms:** "adolescentes"; "jovens"; "adolescência"; "escolares"; "estudantes".

**Spanish language terms:** "adolescentes"; "escolares"; "estudiantes".

**Number of studies identified according to the investigated databases**

**SciELO (total, n = 327)**

**Portuguese language terms:** ((("pressão arterial") OR ("pressão sanguínea") OR ("hipertensão")) AND (("índice de massa corporal") OR ("estado nutricional") OR ("obesidade") OR ("composição corporal") OR ("obesidade abdominal") OR ("circunferência da cintura") OR ("relação cintura-quadril") OR ("razão cintura-estatura") OR ("pregas cutâneas"))) AND (("adolescente")).

**English language terms:** ("blood pressure") OR ("hypertension") AND ("body mass index") OR ("nutritional status") OR ("obesity") OR ("body composition") OR ("abdominal obesity") OR ("waist circumference") OR ("waist-to-hip ratio") OR ("waist-to-height ratio") OR ("skinfolds") AND ("adolescent").

**Spanish language terms:** ("presión arterial") OR ("hipertensión") AND (indice de masa corporal") OR ("estado nutricional") OR ("obesidad") OR ("composición corporal") OR obesidad abdominal") OR (circunferencia de la cintura") OR ("relación cintura-cadera") OR ("relación cintura-altura") OR ("pliegue cutáneo") AND ("adolescente").

**Medline database by PubMed website (total, n = 12.275)**

**Applied filters:** Humans, inglês, portugês, espanhol, birth 18 years.

**English language terms:** ("blood pressure") OR ("hypertension") AND ("body mass index") OR ("nutritional status") OR ("obesity") OR ("body composition") OR ("abdominal obesity") OR ("waist circumference") OR ("waist-to-hip ratio") OR ("waist-to-height ratio") OR ("skinfolds") AND (“adolescent”).

**Scopus (total, n = 1475)**

**Applied filters:** AND NOT (adults) AND NOT INDEX (medline) AND (LIMIT-TO (DOCTYPE, "ar")) AND (LIMIT-TO (LANGUAGE, "English") OR LIMIT-TO (LANGUAGE, "Spanish") OR LIMIT-TO (LANGUAGE, "Portuguese”), TITLE-ABS-KEY**.**

**English language terms:** ( "blood pressure") OR ("hypertension") AND ("body mass index") OR ("nutritional status") OR ("obesity") OR ("body composition") OR ("abdominal obesity") OR ("waist circumference") OR ("waist-to-hip ratio") OR ("waist-to-height ratio") OR ("skinfolds") AND ("adolescent").

**Sportdiscus database by EBSCOhost website (total, n = 144)**

**Applied filters:** all child 0-18 years.

**English language terms:** ("blood pressure") OR ("hypertension") AND ("body mass index") OR ("nutritional status") OR ("obesity") OR ("body composition") OR ("abdominal obesity") OR ("waist circumference") OR ("waist-to-hip ratio") OR ("waist-to-height ratio") OR ("skinfolds") AND (“adolescent”).

**Web of Science (total, n = 1394)**

**Applied filters:** article.

**English language terms:** (("blood pressure" OR "hypertension") AND ("body mass index" OR "nutritional status" OR "obesity" OR "body composition" OR "abdominal obesity" OR "waist circumference" OR "waist-to-hip ratio" OR "waist-to-height ratio" OR "skinfolds") AND ("adolescent")).
