# Supplementary material for: Accuracy of anthropometric indicators of obesity to identify high blood pressure in adolescents—systematic review
Source: PeerJ. 2022 Aug 9;10:e13590. doi: 10.7717/peerj.13590 (PMC9373973; doi:10.7717/peerj.13590)
Supplement: Supplemental Information 3 [file peerj-10-13590-s003.docx]

**Supplementary material 3.** Guidance on the use of the methodological quality/risk of bias assessment instrument for studies of primary diagnostic accuracy (QUADAS-2)

# **Phase 1: State the review question:**

| *Patients (setting, intended use of index test, presentation, prior testing):* |
| --- |
| *Index test(s):* IAO |
| *Reference standard and target condition:* PA |

**Phase 2: Draw a flow diagram for the primary study**

**Phase 3: Risk of bias and applicability judgments**

QUADAS-2 is structured so that 4 key domains are each rated in terms of the risk of bias and the concern regarding applicability to the research question (as defined above). Each key domain has a set of signalling questions to help reach the judgments regarding bias and applicability.

| **DOMAIN 1: PATIENT SELECTION**  **A. Risk of Bias** |  |  |
| --- | --- | --- |
| Describe methods of patient selection: |  |  |
| - Was a consecutive or random sample of patients enrolled? | | Yes/No/Unclear |
| - Was a case-control design avoided? |  | Yes/No/Unclear |
| - Did the study avoid inappropriate exclusions? |  | Yes/No/Unclear |
| **Could the selection of patients have introduced bias?** | **RISK: LOW/HIGH/UNCLEAR** | |
| **B. Concerns regarding applicability** |  |  |
| Describe included patients (prior testing, presentation, intended use of index test and setting)**:** | | |
| **Is there concern that the included patients do not match the review question?** | **CONCERN: LOW/HIGH/UNCLEAR** | |

| **DOMAIN 2: INDEX TEST(S)**  **If more than one index test was used, please complete for each test.**  **A. Risk of Bias** |  |  |
| --- | --- | --- |
| Describe the index test and how it was conducted and interpreted: |  |  |
| - Were the index test results interpreted without knowledge   of the results of the reference standard? | | Yes/No/Unclear |
| - If a threshold was used, was it pre-specified? |  | Yes/No/Unclear |
| **Could the conduct or interpretation of the index test**  **have introduced bias?** | **RISK: LOW/HIGH/UNCLEAR** | |
| **B. Concerns regarding applicability**  **Is there concern that the index test, its conduct, or interpretation differ from the review question?** |  |  |
|  | **CONCERN: LOW/HIGH/UNCLEAR** | |

| **DOMAIN 3: REFERENCE STANDARD**  **A. Risk of Bias** |  |  |
| --- | --- | --- |
| Describe the reference standard and how it was conducted and interpreted: | | |
| - Is the reference standard likely to correctly classify the target   condition? | | Yes/No/Unclear |
| - Were the reference standard results interpreted without knowledge of the results of the index test? | | Yes/No/Unclear |
| **Could the reference standard, its conduct, or its**  **interpretation have introduced bias?** | **RISK: LOW /HIGH/UNCLEAR** | |
| **B. Concerns regarding applicability** |  |  |
| **Is there concern that the target condition as defined by the reference standard does not match the review question?** | **CONCERN: LOW/HIGH/UNCLEAR** | |

| **DOMAIN 4: FLOW AND TIMING**  **A. Risk of Bias** |  |  |
| --- | --- | --- |
| Describe any patients who did not receive the index test(s) and/or reference standard or who were excluded from the 2x2 table (refer to flow diagram):  Describe the time interval and any interventions between index test(s) and reference standard: | | |
| - Was there an appropriate interval between index test(s)   and reference standard? | | Yes/No/Unclear |
| - Did all patients receive a reference standard? |  | Yes/No/Unclear |
| - Did patients receive the same reference standard? |  | Yes/No/Unclear |
| - Were all patients included in the analysis? |  | Yes/No/Unclear |
| **Could the patient flow have introduced bias?** | **RISK: LOW /HIGH/UNCLEAR** | |
